# Supplementary material for: Transcriptome Analysis Reveals Vimentin-Induced Disruption of Cell–Cell Associations Augments Breast Cancer Cell Migration
Source: Cells. 2022 Dec 13;11(24):4035. doi: 10.3390/cells11244035 (PMC9776984; doi:10.3390/cells11244035)
Supplement: Supplementary file 1 [file cells-11-04035-s001.zip › DEGs and enrichment analysis.zip!/DEGs and enrichment analysis/WT_VIMvsVector KEGG/DOWN/WT_VIMvsVector_DOWN.pathway_map.zip!/WT_VIMvsVector_DOWN.pathway_map/src/hsa04621.html]

hsa04621


- hsa:2634

- Down regulated genes

ENSG00000162645(-1.85360634870597)

- hsa:2635

- Down regulated genes

ENSG00000117226(-2.72954644386849)
- hsa:2634

- Down regulated genes

ENSG00000162645(-1.85360634870597)

- hsa:330

- Down regulated genes

ENSG00000023445(-2.12138419902495)

- hsa:23710

- Down regulated genes

ENSG00000139112(-1.85791559298273)

- hsa:84674

- Down regulated genes

ENSG00000132357(-3.12592181354519)

- hsa:4939

- Down regulated genes

ENSG00000111335(-2.07102499896395)

- hsa:3576

- Down regulated genes

ENSG00000169429(-3.18264312774765)

- hsa:2920

- Down regulated genes

ENSG00000081041(-2.33759639518744)

Close
